# Barriers and facilitators to access and uptake of intermittent preventive treatment with sulfadoxine-pyrimethamine among pregnant women in Nigeria: a scoping review

**Published:** 2022-06-21

**Authors:** Patricia Ogba, Andrea Baumann, Hanna Chidwick, Laura Banfield, Deborah D. DiLiberto

**Affiliations:** 1 Faculty of Health Sciences, Global Health Office, McMaster University, Main St. W, Hamilton, Ontario, Canada; 2 Health Sciences Library, McMaster University, Main St. W, Hamilton, Ontario, Canada

## Abstract

**Background:**

Malaria in pregnancy is a significant public health concern in Nigeria. It threatens pregnant women and their unborn babies and undermines the achievement of Sustainable Development Goal 3. The World Health Organization has recommended intermittent preventive treatment with sulfadoxine-pyrimethamine [IPTp-SP] for its control, but there are challenges to its access and uptake.

**Methods:**

Using the Arksey and O'Malley framework and the cascade of care model, we conducted a scoping review to investigate barriers and facilitators of IPTp-SP access and uptake, including their influence on pregnant women's health-seeking behaviour for the control of malaria in pregnancy in Nigeria. We searched seven scientific databases for papers published from 2005 to date.

**Results:**

We included a total of 31 out of 2149 articles in the review. Poor provider knowledge of the IPTp-SP protocol and lack of essential commodities for sulphadoxine-pyrimethamine administration in clinics are significant barriers to IPTp-SP use. Staff shortages and poor remuneration of health care professionals are obstacles to IPTp-SP utilisation.

**Conclusions:**

To improve IPTp-SP access and uptake, the government should ensure a continuous supply to clinics and support the employment of additional health care professionals who should be well paid and trained on using the IPTp-SP protocol.

## Introduction

Malaria in pregnancy [MiP] is one of the major causes of maternal mortality and adverse pregnancy outcomes in Nigeria. It undermines the achievement of Sustainable Development Goal 3, which targets maternal mortality reduction and malaria eradication by 2030 [1-3]. Fortunately, chemoprophylaxis can help to prevent MiP [1]. The World Health Organization (WHO) recommends that all pregnant women in malaria-endemic areas receive at least three rounds of intermittent preventive treatment with sulfadoxine-pyrimethamine (IPTp-SP) for MiP control. Despite adopting this guideline, the uptake of IPTp-SP among pregnant women in many African countries remains very low [4,5].

The Federal Ministry of Health in Nigeria introduced the IPTp-SP strategy in 2005. As a preventative treatment, IPTp-SP is given to pregnant women, whether they have malaria or not, at monthly intervals from the second trimester until delivery. Under the national protocol, IPTp-SP is offered free as a directly observed treatment (DOT) through ante-natal clinics (ANCs) in public hospitals and non-governmental organisation facilities [6-8]. However, challenges at all levels of the health system, including macro-level issues and supply and demand issues, limit the effectiveness of this policy [1,5,9,10] and impede the progress on MiP control in Nigeria. The percentage of women who receive one IPTp-SP round has increased steadily since 2014, but uptake of two and three rounds remains low [11]. In 2018, Nigeria had one of the highest occurrences of MiP, but less than 25% of ANC attendees completed the recommended three rounds [7,12]. With about 90% of Nigeria’s 7.5 million pregnant women being at risk of getting malaria annually [1,13], an incomplete course of IPTp-SP can have severe consequences for women and their babies. Malaria accounts for almost 50% of the diseases and for 15% of anaemia reported among pregnant women in Nigerian hospitals. Reports of morbidity and mortality among pregnant women due to this disease are estimated to be 70.5% and 11%, respectively. Among neonates, the incidence of between 5-14% low birth weight (LBW) and about 30% preventable LBW is attributed to MiP [2,5,10,14-19]. The failure to achieve broad coverage of IPTp-SP among pregnant women reflects the acknowledgement of a gap between an available policy and the barriers to its implementation.

IPTp-SP acceptance and coverage have been an investigation target for over ten years [4,20-22]. However, to the best of our knowledge, no single review focussing on Nigeria has been conducted. Using elements of the Cascade of Care model (CoC), this study examined the barriers and facilitators of IPTp-SP access and uptake, including their influence on pregnant women's health-seeking behaviour for MiP control in Nigeria. The CoC, which has been used successfully in chronic communicable and non-communicable disease programmes [23-26], describes the care-seeking steps of patients from diagnosis to treatment. It helps to analyse patients' progress towards achieving better health outcomes and tackling any identified potential barriers to seeking care [27]. The CoC is a valuable framework for exploring the obstacles and facilitators along the pathway of IPTp-SP utilisation for MiP control. We anticipate that the study findings will inform future research into the challenges facing IPTp-SP coverage and improve its utilisation across Nigeria.

## Materials and Methods

We used the Arksey and O'Malley [28] methodological framework and the Preferred Reporting Items for Systematic Reviews and Meta-Analyses extension for Scoping Reviews (PRISMA-ScR) checklist by Tricco *et al.* [29] (see checklist in Appendix A). A five-stage process was used: (1) identifying the research question, (2) identifying relevant studies, (3) study selection, (4) charting the data, and (5) collating, summarising and reporting results [28]. A scoping review protocol was created and is available upon request.

This review adapted the CoC model with the following key stages: (a) a woman is diagnosed with pregnancy, (b) pregnant woman registers in an ANC and attends ANC regularly, (c) pregnant woman takes IPTp-SP at least three times during pregnancy.

This pathway shows that upon becoming pregnant, women should register in and be consistent with ANC visits to get the three or more IPTp-SP rounds required to protect them from MiP. However, several barriers along this pathway span throughout and beyond the health system, including individual and interpersonal factors.

### Identifying the research question

This review was guided by the research question: "What are the barriers and facilitators to IPTp-SP utilisation, and their influence on the health-seeking behaviour for the control of MiP among pregnant women in Nigeria?”.

### Identifying relevant studies

We developed search strategies in collaboration with an experienced librarian at a post-secondary institution. Seven databases were searched through CINAHL, Embase, Web of Science, MEDLINE, Global Health, JSTOR, and Malaria in Pregnancy. The search was limited to English language articles because of the language skills of team members and to publications from 2005 to the present because the IPTp-SP policy was adopted in Nigeria that same year. The searches were conducted from January to March 2021, with the most recent search executed on March 20, 2021. See Appendix B for the complete search strategies.

### Inclusion and exclusion criteria

We included articles focused on IPTp-SP access, coverage, uptake, barriers, facilitators, and utilisation in Nigeria. Books or chapters and articles focused on other antimalarials, children and infants, HIV positive and sickle cell anaemic pregnant women were excluded.

### Study selection

Two reviewers (PO and HC) screened the study titles, abstracts, and full texts using Covidence, a web-based reviewing platform. The two reviewers discussed disagreement about study eligibility until consensus.

### Charting the data

A charting template, shown in Table 1, was used to record relevant information from included articles [30].

**Table 1.** Data extraction template.

S/N	Variable	Description
1	Authors	Names of authors that published paper
2	Year of publication	Year in which paper was published
3	Title	Title of paper
4	Aims/objectives	The aims/objectives that guided the conduct of the study
5	Study design	Type of study design
6	Study population	Participants of the study
7	Key findings	Information from the study relevant to the review question/objective

## Results

**Figure 1.**
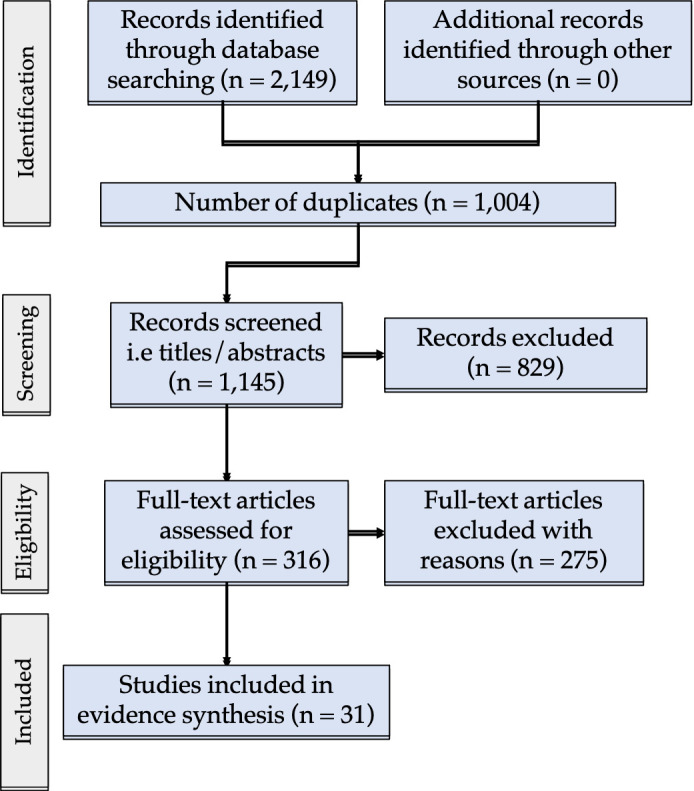
PRISMA flow chart for analysed studies

A total of 2149 articles were exported to Covidence. After duplicates were removed, the titles and abstracts of 1145 articles were screened. Next, 316 full-text articles were screened, and 31 studies were included in the review (Figure 1). Articles were excluded during the full-text screening phase for the following reasons:

- Non-availability of the full text.
- The studies focused on populations other than pregnant women.
- Another source better covers information.
- Studies covered sub-Saharan African countries other than Nigeria.

**Figure 2.**
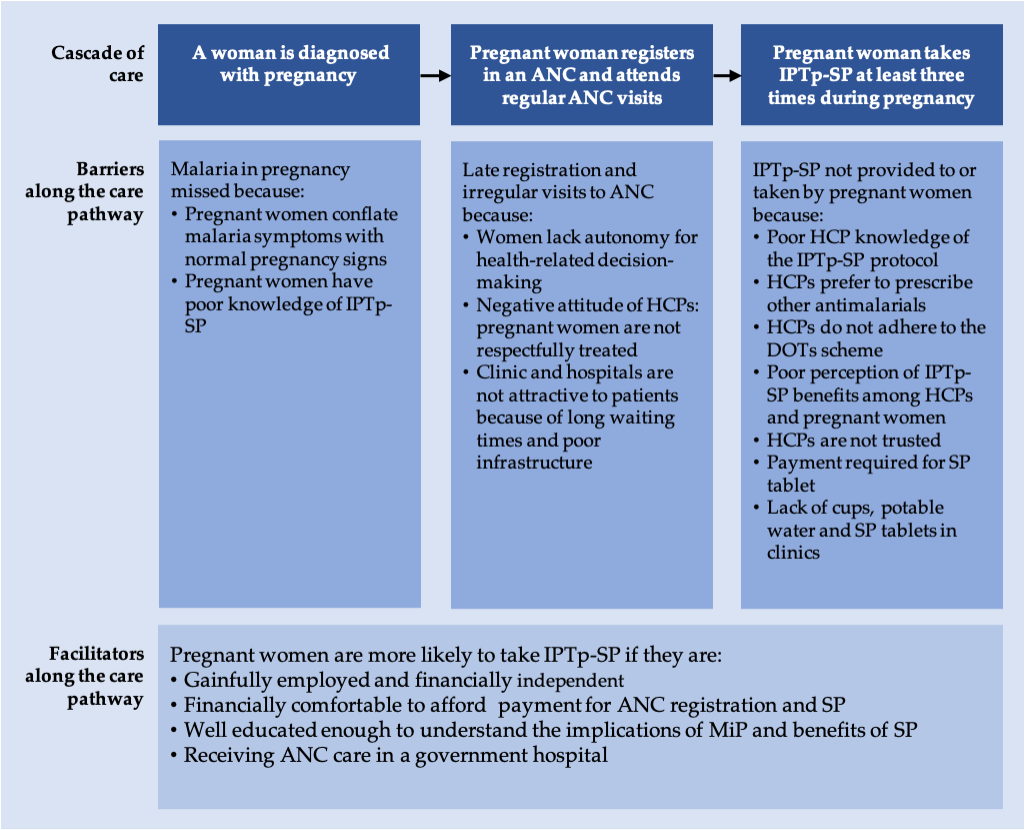
Barriers and Facilitators of IPTp-SP utilization along the Cascade of Care

The adapted CoC model was used to categorise and examine the barriers and facilitators of IPTp-SP access and uptake in the literature (Figure 2).

### Woman diagnosed with pregnancy

In this phase, individual-level factors such as perception about pregnancy and poor knowledge of IPTp-SP act as barriers to IPTp-SP utilisation.

Generally, pregnancy is seen as a regular occurrence and not an illness. While this is correct, it prevents pregnant women and their spouses from accepting medical interventions like IPTp-SP [31]. People also consider some malaria symptoms like high fever and general weakness typical pregnancy signs. This belief affects care-seeking behaviour for the control and treatment of MiP [32].

Similarly, poor knowledge of IPTp-SP prevents pregnant women from requesting SP in clinics; furthermore, in instances where they get the drug outside the hospital, it may be inappropriately used [33,34]. Many pregnant women report that ANC providers do not give enough details about IPTp-SP, including the required dose and timing, benefits, or why it is important in pregnancy. Some women refuse the treatment, thinking that only individuals with malaria should take IPTp-SP [2,7,35-37].

### Pregnant woman registers in an ANC and attends ANC regularly

After pregnancy confirmation, a woman should register in an ANC where IPTp-SP is prescribed and administered. However, individual, interpersonal, and health system factors impede IPTp-SP utilisation.

The WHO recommends at least eight ANC visits during pregnancy to enhance pregnant women's chances of completing three or more rounds of IPTp-SP [37,38]. The literature, however, shows that most women do not register, register late, or are irregular with ANC visits, preventing them from getting or completing the required IPTp-SP dose before delivery [20,38-41]. Within the Nigerian culture, women lack the autonomy to make health-related decisions independent of their husbands and sometimes sisters and mothers-in-law. There have been reports of women not attending ANC or taking IPTp-SP because their spouses did not consent [2,7,10,31,33,36]. Some men discourage ANC visits because of cost, while others do so because they prefer traditional birth attendants (TBAs) [31].

Delays and the negative attitude of healthcare professionals (HCPs) discourage pregnant women from regularly attending ANC, especially in public hospitals where they can obtain free SP [7,31]. They explore other options, including self-medication with herbal mixtures and unprescribed antimalarials, and seek treatment from traditional and faith homes, where they claim to be respectfully treated [7,31,32]. The unfriendliness of HCPs has been attributed to lack of job satisfaction, low remuneration, poor work environment, and excess workload; however, long wait times are attributed to understaffing [1,31,42].

Facilitators of IPTp-SP utilization include employment status, wealth index, and level of education. Gainfully employed pregnant women and those in the higher wealth index are more likely to register in an ANC and utilise IPTp-SP because they can make financial and health-related decisions independent of their spouses [1,10,38,39,43-45]. Similarly, pregnant women who are educated and have educated spouses are more likely to attend ANC and use IPTp-SP. Education has been shown to enhance pregnant women's understanding of the adverse effects of malaria and the importance of its preventive strategies [36,38,40,44-46].

### Pregnant woman takes IPTp-SP at least three times during pregnancy

HCP unawareness of the availability and implementation of the IPTp-SP protocol is a challenge at this stage of the cascade. Some private hospitals do not have this protocol as part of their treatment policy [2,19,22,37,47-50]. Physicians still prescribe the weekly regimen of chloroquine and pyrimethamine despite their reduced potency and poor compliance among pregnant women. Reasons cited for this prescription style are the affordability and accessibility of other antimalarials and poor knowledge of drug resistance patterns among physicians [6,22,31,47,50]. Only a few HCPs, mostly in public hospitals, know the IPTp-SP protocol, but there is confusion regarding dose, timing, and the gestational age to commence treatment. Insufficient training and supervision of HCPs are highlighted as reasons for this poor understanding [2,19,22,48-50].

Furthermore, some pregnant women refrain from IPTp-SP because they think it could cause harm, especially in the second and third trimesters. They report that the drug weakens them and associate its use with frequent urination and adverse outcomes like skin reactions, abortions, and foetal abnormalities. Some women consider taking SP a waste of time because they doubt its efficacy [2,7,20,34,37,51]. Others refuse because the drug is unfamiliar, and their husbands did not buy it. This shows a distrust for HCPs which may be linked to previous rumours in the region about how vaccines were reportedly fortified with anti-fertility drugs to prevent conception [31,36].

Under the IPTp-SP scheme in Nigeria, SP is free as it is supplied to public clinics by the government. Unfortunately, this drug is frequently unavailable because the government fails to ensure its constant supply [19,20,31]. Pregnant women either are turned away without receiving IPTp-SP or are asked to pay for it. Prescription notes are sometimes given to pregnant women to buy the drug from pharmacies without the assurance that the right medicine will be purchased or used appropriately [2,7]. User fees impose additional costs on pregnant women, who are usually disappointed and often refuse to buy SP because they know it should be free [19,49]. Unavailability of potable water and clean cups to administer SP as a directly observed treatment (DOT) and inadequate staff to monitor pregnant women taking the drug also limit pregnant women's uptake of complete IPTp-SP rounds [19,35,39,49]. Other issues include HCP unawareness of IPTp-SP as a DOT and the safety of using SP without food. Some HCPs have told pregnant women to use SP at home after meals, unknowingly giving these women the opportunity to default with drug uptake [33,49]. Further, despite the convergence of evidence demonstrating the benefits and safety of using IPTp-SP, some HCPs in Nigeria still think contrarily. Consequently, HCPs minimally prescribe IPTp-SP, preventing pregnant women from taking at least three rounds of IPTp-SP [4,7,37,44,48-50].

Facilitators in this phase are the use of government hospitals and geographical locations. Government hospitals have reported increased IPTp-SP utilisation due to HCPs educating ANC attendees about the benefits [6,10,43]. Making SP available free of charge in government hospitals also increases IPTp-SP uptake [6,10,43,47]. Adeola and Okwilagwe [10] discovered that rural pregnant women are better users of IPTp-SP than their urban counterparts. Their receptiveness may be connected to the Rollback Malaria [RBM] programme, which enhances IPTp-SP prescription and focuses more on rural communities in Nigeria. Anecdotal evidence suggests that urban dwellers make minimal effort to prevent MiP because they see it as a common disease [10]. Contrastingly, Ndu *et al.* [43] and Olugbade *et al.* [44] posit that urban pregnant women are better users of IPTp-SP because they can easily access service providers and current information from the media. Oyefabi *et al.* [19] found that IPTp-SP is more readily available in urban health centres. More research needs to be done to resolve these contrasting views.

## Discussion

Despite the availability of a clear policy and the known effectiveness of IPTp-SP in preventing MiP, uptake and completion of the recommended dose by pregnant women remains very low [1]. Most barriers to IPTp-SP utilisation are within the health system, but individual and interpersonal factors are also significant obstacles [1,2,4,52]. Our study highlights the interconnection between the health system, individual, and interpersonal barriers and their influence on one another. For example, frequent SP shortage has resulted in the introduction of user fees (health system factors), which discourages pregnant women from attending ANCs (individual factor) and encourages their loss of trust in HCPs (interpersonal factor) because they know SP should be free [31,32,49].

For IPTp-SP utilisation to improve in Nigeria, gaps within the health system must be addressed [1]. First, the government must provide continuous training for all HCPs regarding the IPTp-SP protocol to improve their knowledge of its benefits, reduce their concerns about risks, and encourage its prescription. Second, supportive supervision of HCPs by credible peers is needed to promote IPTp-SP policy implementation and guarantee proper quality assurance and monitoring, ultimately improving IPTp-SP utilisation [1,4,53]. Third, increased government commitment towards providing SP, potable drinking water, and clean cups is required and has been shown to increase IPTp-SP coverage and uptake in other countries, including Zambia and Senegal [4,53,54]. There are also records of how these approaches have eliminated SP user fees and enhanced HCP adherence to the DOTs strategy recommended for IPTp-SP administration [2,19,20,31,33,35,39,49,55].

The encouragement of HCPs is likewise vital [1]. Many HCPs in Nigeria are dissatisfied with their jobs and limited career development opportunities. They are also overworked and inadequately remunerated [1,7,31,42]. Studies have found that overworked and underpaid staff may be hostile towards clients, and this hostility negatively influences clients' health-seeking behaviour [42,53]. Efforts should be made to hire more HCPs, increase their pay, and provide career enhancement and educational opportunities.

Individual factors also need to be addressed. Pregnant women's poor knowledge and understanding of the use and benefits of IPTp-SP affect their perception of IPTp-SP safety and efficacy, erroneously linking its use to abortion. This flawed perception often prevents pregnant women from demanding and using the drug [2,7,34-37,51,55]. While mild and brief side effects like nausea, vomiting, and dizziness may occur when using SP the first time, this drug is generally well tolerated [56]. Health education can increase uptake by enhancing understanding of the benefits and safety of SP and correcting some of the misconceptions about pregnancy that prevent pregnant women from accepting medical interventions [2,4].

Considering the success of community mobilisation with the onchocerciasis control programme in Uganda, a similar strategy may be effective for promoting and augmenting ANC attendance and IPTp-SP utilisation [2,4,53]. A community mobilisation effort involves pregnant women's access to SP, frequent personalised health education, follow-up visits and reminders from Community Directed Distributors (CDD), even when they miss ANC. This programme does not undermine ANC attendance because the CDDs are trained to refer pregnant women to hospitals for complete ANC services [2,53,57,58]. This cost-effective and feasible strategy has demonstrated positive results in studies in Uganda [59,60]. In addition, employing a group ANC service delivery model could encourage more robust peer support for and administration of IPTp-SP through increased ANC visits [61].

The interpersonal factor of cultures is a challenge because it is a way of life passed down through generations and is difficult to change. Since the Nigerian culture supports that men make health-related decisions for their pregnant wives, a practical approach may be to educate these men on the dangers of MiP and the benefits of IPTp-SP [4,31,36]. Traditional and faith-based providers should receive similar education and training, as some women are permitted to seek ANC services only from these groups. Adequate knowledge of MiP, IPTp-SP use, and where it can be accessed can encourage traditional birth attendants and faith-based providers to refer their clients to public hospitals for MiP control [7]. Regarding pregnant women's lack of trust in HCPs and men discouraging their wives from ANC visits because of SP payment, eradication of user fees through constant SP availability may resolve this [4,53]. Finally, training HCPs to provide customer service respectfully may increase the likelihood that pregnant women will comply with IPTp-SP uptake in clinics [31,53].

## Recommendations

A comprehensive approach is required to overcome barriers and mitigate their individual and collective impact. It is recommended that user fees be eliminated through regular SP availability and that HCP understanding of adherence to the national IPTp-SP protocol is increased [2,19,20,31,33,35,39,49,55]. Hiring more HCPs can also help to alleviate their stress and reduce their hostility toward their patients. Furthermore, the distribution of SP should not be limited to clinics but should be offered to pregnant women in the community. This approach can reach women, particularly those in rural areas, who do not visit the ANC during their pregnancy [20,57,58]. Finally, adopting a group antenatal clinic service approach may give pregnant women the opportunity to build social support, encouraging IPTp-SP use [61,62].

## Limitations

Only one reviewer extracted data. However, two reviewers screened and identified relevant studies using pre-determined inclusion and exclusion criteria to minimise error. Due to financial and time constraints, grey literature and consultation with key stakeholders were excluded despite being suggested by the framework used [28]. Although the literature search was limited to English articles, the diversity of journals included contributes to the strength of the review.

## Conclusion

This review has shown how barriers at the individual, interpersonal, and health system levels affect implementing the IPTp-SP policy and its uptake. If Nigeria is to achieve SDG3 and reach the much-desired reduction in MiP burden, the identified bottlenecks must be tackled. The review highlights the need for increased government involvement and investments in hiring, training, and financially supporting HCPs. The availability of tools for IPTp-SP administration in clinics should also be ensured. Most of the papers included in this review focused on health system barriers to the uptake of IPTp-SP, making it apparent that there is a lack of studies on how factors within the community influence IPTp-SP uptake among pregnant women in Nigeria. Further research is therefore needed to understand how community-level contextual factors influence pregnant women's uptake of recommended IPTp-SP doses.

## Appendix A

Below is the completed Preferred Reporting Items for Systematic reviews and Meta-Analyses extension for Scoping Reviews (PRISMA-ScR) checklist, retrieved from Tricco *et al.* [29].

**Table:** PRISMA-ScR Checklist

Section	Item	Prisma-scr * checklist item	Reportedon page #
**Title**	1	Identify the report as a scoping review.	1
**Abstract**			
Structured summary	2	Provide a structured summary that includes (as applicable) background, objectives, eligibility criteria, sources of evidence, charting methods, results, and conclusions that relate to the review questions and objectives.	1
**Introduction**			
Rationale	3	Describe the rationale for the review in the context of what is already known. Explain why the review questions/objectives lend themselves to a scoping review approach.	1-2
Objectives	4	Provide an explicit statement of the questions and objectives being ad-dressed with reference to their key elements (e.g., population or participants, concepts, and context) or other relevant key elements used to conceptualize the review questions and/or objectives.	2
**Methods**			
Protocol and regi-stration	5	Indicate whether a review protocol exists; state if and where it can be ac-cessed (e.g., a Web address); and if available, provide registration infor-mation, including the registration number.	Available on request
Eligibility criteria	6	Specify characteristics of the sources of evidence used as eligibility criteria (e.g., years considered, language, and publication status), and provide a rationale.	2
Information sources*	7	Describe all information sources in the search (e.g., databases with dates of coverage and contact with authors to identify additional sources), as well as the date the most recent search was executed.	2
Search	8	Present the full electronic search strategy for at least 1 database, including any limits used, such that it could be repeated.	Appendix
Selection of sources of evidence	9	State the process for selecting sources of evidence (i.e., screening and eli-gibility) included in the scoping review.	2
Data charting process	10	Describe the methods of charting data from the included sources of evi-dence (e.g., calibrated forms or forms that have been tested by the team before their use, and whether data charting was done independently or in duplicate) and any processes for obtaining and confirming data from investigators.	3
Data items	11	List and define all variables for which data were sought and any assump-tions and simplifications made.	3
Critical appraisal of individual sources of evidence	12	If done, provide a rationale for conducting a critical appraisal of included sources of evidence; describe the methods used and how this information was used in any data synthesis (if appropriate).	N/A
Summary measures	13	Not applicable for scoping reviews.	N/A
Synthesis of results	14	Describe the methods of handling and summarizing the data that were charted.	3
Risk of bias across studies	15	Not applicable for scoping reviews.	N/A
Additional analyses	16	Not applicable for scoping reviews.	N/A
**Results**			
Selection of sources of evidence	17	Give numbers of sources of evidence screened, assessed for eligibility, and included in the review, with reasons for exclusions at each stage, ideally using a flow diagram.	3
Characteristics of sources of evidence	18	For each source of evidence, present characteristics for which data were charted and provide the citations.	Appendix C
Critical appraisal within sources of evidence	19	If done, present data on critical appraisal of included sources of evidence (see item 12).	N/A
Results of individual sources of evidence	20	For each included source of evidence, present the relevant data that were charted that relate to the review questions and objectives.	Appendix C
Synthesis of results	21	Summarize and/or present the charting results as they relate to the re-view questions and objectives.	3-5
Risk of bias across studies	22	Not applicable for scoping reviews.	N/A
Additional analyses	23	Not applicable for scoping reviews.	N/A
**Discussion**			
Summary of eviden-ce	24	Summarize the main results (including an overview of concepts, themes, and types of evidence available), link to the review questions and objectives, and consider the relevance to key groups.	5-6
Limitations	25	Discuss the limitations of the scoping review process.	7
Conclusions	26	Provide a general interpretation of the results with respect to the review questions and objectives, as well as potential implications and/or next steps.	7
**Funding**	27	Describe sources of funding for the included sources of evidence, as well as sources of funding for the scoping review. Describe the role of the funders of the scoping review.	N/A

*From: Tricco AC, Lillie E, Zarin W, O'Brien KK, Colquhoun H, Levac D, et al. PRISMA extension for scoping reviews (PRIS-MA-ScR): Checklist and explanation. Ann Intern Med. 2018;169(7):467–73.*

## Appendix B

To generate search terms, the research question was broken down into keywords. From these keywords, a large list of synonyms and related terms were identified, using as many terms as possible. Synonyms and related terms for each category were combined using Boolean operators "OR" and "AND".

In complying with the PRISMA-ScR checklist, the search query for the database OVID is provided below:(exp pyrimethamine plus sulfadoxine/) OR (IPTp-SP.mp.) OR (exp fansidar/) OR (intermittent preventive treatment.mp.) OR (intermittent preventive therapy.mp.) OR (intermittent presumptive treatment.mp.) AND (exp placenta malaria/) OR (exp malaria falciparum/) OR (malaria.mp.) OR (exp malaria/) AND (exp pregnancy/) OR (pregnancy.mp.) OR (pregnant women.mp.) OR (exp pregnant woman/) OR (pregnant.mp.) AND (Nigeria.mp.) OR (exp Nigeria/) OR (sub-Saharan Africa.mp.) OR (exp “Africa south of the Sahara”/)

## Appendix C

**Table:** The characteristics of the studies included in this review are listed Included literature after a systematic search

Author(s) (Year)	Reference number	Title	Aims/Objectives	Study design/population	Key findings
Adeniran, Mobolaji- Ojibara, Adesina, Aboyeji, Ijaiya, Balogun (2018)	5	Intermittent preventive therapy in pregnancy with sulfadoxine/pyrimethamine for malaria prophylaxis among parturients in Ilorin, Nigeria	To assess the knowledge, attitude, and factors associated with the use of IPTp-SP among antenatal clinic attendees in Ilorin	Quantitative / Pregnant women	Parturients desire to use IPTp-SP was hampered by HCPs attitude of prescribing other antimalarial, misconceptions about drug safety, and frequent shortage of SP in clinics
Adeola, & Okwilagwe (2015)	10	Acceptance and Utilization of Sulfadoxine-Pyrimethamine and Insecticide-Treated Nets among Pregnant Women in Oyo State, Nigeria	To investigate the acceptability and utilization of RBM tools, their influencing factors, and the influence of location on the behaviour outcomes among pregnant women who access government and faith clinics in Oyo State, Nigeria	Quantitative / Pregnant women	The acceptance of RBM tools, including IPTp-SP, though still far from meeting the set targets, was higher among rural pregnant women who accessed government clinics than among urban pregnant women who accessed faith clinics
Adewole, Fawole, Ajayi, Yusuf, Oladimeji, Waziri, Nguku, Ajumobi (2019)	39	Determinants of intermittent preventive treatment of malaria among women attending antenatal clinics in primary health care centers in Ogbomoso, Oyo State, Nigeria	To assess the factors influencing the utilization of intermittent preventive treatment in pregnancy in Ogbomoso, Oyo State, Nigeria	Mixed methods / Pregnant women and healthcare workers	IPTp-SP uptake was low because of late ANC booking and frequent SP stock-outs
Akinleye, Falade, Ajayi (2009)	20	Knowledge and utilization of intermittent preventive treatment for malaria among pregnant women attending antenatal clinics in primary health care centers in rural southwest, Nigeria: a cross- sectional study	To assess the use of IPTp among pregnant women attending primary health centres in the rural area and determine factors that influence the uptake	Quantitative / Pregnant women	IPTp-SP uptake was low because of pregnant women's low awareness and knowledge level on IPTp-SP, late enrolment for ANC, HCPs' poor adherence to the DOTs scheme, and periodic shortage of SP in the clinic
Akpa, Akinyemi, Umeokonkwo, Bamgboye, Dahiru, Adebowale, Ajayi (2019)	40	Uptake of intermittent preventive treatment for malaria in pregnancy among women in selected communities of Ebonyi State, Nigeria	To determine the level of IPTp uptake and factors influencing it among women of reproductive age residing in communities in Ebonyi State	Quantitative / Women of reproductive age	IPTp-SP uptake among women in their last pregnancy was below WHO recommendation, and the husband's education was a determinant of IPTp-SP uptake. Male education is recommended as this may positively influence IPTp-SP uptake
Ameh, Owoaje, Oyo- Ita, Kabiru, Akpet, Etokidem, Enembe, Ekpenyong (2016)	33	Barriers to and determinants of the use of intermittent preventive treatment of malaria in pregnancy in Cross River State, Nigeria: a cross- sectional study	To identify the barriers to and determinants of the use of IPTp-SP among pregnant women attending ANC in PHC facilities in Cross River State, south-south region of Nigeria	Quantitative / Pregnant women	Awareness level and non-compliance of PHC to treatment guidelines may hamper efforts to reduce malaria-related child and maternal morbidity and mortality. In addition to enhancing the capacity of clinics to implement the IPTp- SP guidelines, health education programs on malaria prevention targeting mothers, household heads, and HCPs are needed
Amoran, Ariba, Iyaniwura (2012)	21	Determinants of intermittent preventive treatment of malaria during pregnancy (IPTp) utilization in a rural town in Western Nigeria	To assess the prevalence and determinants of intermittent preventive treatment of malaria (IPTp) utilization by pregnant women in a rural town in Western Nigeria	Quantitative / Pregnant women	The uptake of IPTp-SP among rural ANC attendees was low due to poor knowledge of prophylaxis for malaria prevention. Community health education may help to improve IPTp-SP uptake
Arulogun, Okereke (2012)	50	Knowledge and practices of intermittent preventive treatment of malaria in pregnancy among health workers in a southwest local government area of Nigeria	To assess the level of knowledge and practice of IPTp among health workers in Ibadan North Local Government Area, Nigeria	Mixed methods / Pregnant women and health workers	Poor knowledge of IPTp and poor compliance to DOTs are significant bottlenecks to IPTp implementation: There is a need for health promotion and education intervention targeting health workers
Bello, & Oni (2020)	48	Health Workers' Awareness and Knowledge of Current Recommendation of Intermittent Preventive Treatment in Pregnancy in South-Western Nigeria	To assess health worker's awareness and knowledge of the current World Health Organization (WHO) recommendation of intermittent preventive treatment in pregnancy with sulfadoxine-pyrimethamine (IPTp-SP)	Quantitative / Health workers	HCPs poor knowledge of the correct administration of IPTp-SP, poor adherence to the current WHO IPTp-SP recommendation, and the attitude of prescribing other antimalarials was contributing to the increasing prevalence of MiP and its complications
Chukwurah, Idowu, Adeneye, Aina, Agomo, Otubanjo (2016)	34	Knowledge, attitude and practice on malaria prevention and sulfadoxine- pyrimethamine utilization among pregnant women in Badagry, Lagos State, Nigeria	To assess the knowledge, attitude, and practices of pregnant women on malaria prevention, to assess their knowledge of SP for IPT in pregnancy, and to use the outcomes for the creation of awareness on malaria prevention with IPTp-SP among pregnant women or women of child-bearing age	Quantitative / Pregnant women	The majority of pregnant women knew SP but did not know it could prevent malaria. They prevented malaria by taking herbs but went to the hospital for malaria treatment
Dairo, Adekunle, Okedare (2019)	38	Uptake of Intermittent Preventive Treatment for malaria in Nigeria	To describe the uptake of malaria preventive measures recommended by WHO among pregnant women. To determine the pattern in proportion of pregnant women who used IPTp- SP and to identify the factors affecting uptake in Nigeria.	Quantitative / women	The uptake of IPTp-SP is still low and calls for intensified improvement of awareness
Diala, Pennas, Marin, Belay (2013)	31	Perceptions of intermittent preventive treatment of malaria in pregnancy (IPTp) and barriers to adherence in Nasarawa and Cross River States in Nigeria	To identify pregnant women's and providers' perceptions of IPTp, and barriers to adherence to the protocol of at least two doses.	Mixed methods / Pregnant women and health care providers	Many structural barriers are preventing ANC attendees from completing IPTp- SP. Programs with specific interventions to increase IPTp-SP uptake and adherence targeting women, their communities, and the health environment may be helpful
Edet, Edet, Samson- Akpan, Ojong (2013)	35	Missed opportunities for intermittent preventive treatment among pregnant women, in a secondary health facility, Cross River State, Nigeria	To determine the magnitude and contributory factors for missed opportunities for the administration of IPTp during pregnancy among pregnant women attending a secondary health facility in Calabar	Quantitative / Pregnant and postnatal women	Non-availability of SP, lack of supervision, failure to prescribe medication, late booking, and lack of knowledge were responsible for low IPTp-SP uptake. The regular supply of SP is critical to increasing IPTp-SP uptake
Esu, Effa, Udoh, Oduwole, Odey, Chibuzor, Oyo-Ita, Meremikwu (2013)	41	Utilization of intermittent preventive treatment for malaria among pregnant women attending antenatal clinics in health facilities of Cross River State, Nigeria	To assess the utilization of intermittent preventive treatment with sulfadoxine-pyrimethamine for the prevention of malaria in pregnancy against the national treatment policy among women attending health care facilities in Cross River State, Nigeria	Quantitative / Pregnant women	Early and regular ANC visits may be a potential means of increasing IPTp-SP utilization
Harrison, Olufunlayo, Odunukwe (2016)	47	Improved prescription of Intermittent preventive therapy for malaria in pregnancy (IPTp) among doctors practicing in 68 Nigerian army reference hospital Yaba Lagos, Nigeria	To assess the prescription pattern of intermittent preventive therapy with sulphadoxine-pyrimethamine for pregnant women among doctors practicing in 68 Nigeria Army Reference Hospital, Yaba, Lagos, Nigeria.	Quantitative / Doctors	Education of all HCPs in both public and private clinics on MiP prevention guidelines is needed to increase IPTp-SP coverage
Ikpeama, Ikpeama, Ikpeama, Ogwuegbu (2017)	51	Knowledge, attitude and utilization of intermittent preventive treatment for malaria among pregnant women attending antenatal clinic in Usmanu Danfodiyo University Teaching Hospital (UDUTH) Sokoto	To assess the knowledge, attitude, and the uses of intermittent preventive treatment of malaria during pregnancy among pregnant women attending antenatal care visit and determine the factor influencing IPTp and its effectiveness	Quantitative / Pregnant women	Respondents' knowledge and attitude towards IPTp-SP for malaria control were good, but utilization was low due to misconceptions about IPTp-SP and non- adherence of DOTS strategy in clinics.
Iliyasu, Gajida, Galadanci, Abubakar, Baba, Jibo, Aliyu (2012)	36	Adherence to intermittent preventive treatment for malaria in pregnancy in urban Kano, northern Nigeria	To assess the correlates of IPTp use, in addition to knowledge and attitudes to IPTp among women attending urban primary health care centres in Kano	Quantitative / Pregnant women	Health system factors, including demand and supply side contributed to low IPTp- SP adherence and uptake. Health system strengthening through community mobilization, male partner involvement, and community-based distribution of SP may help to overcome these challenges
Muhammad, Majdzadeh, Nedjat, Sajadi, Parsaeian (2020)	45	Socioeconomic inequality in intermittent preventive treatment using sulphadoxine- pyrimethamine among pregnant women in Nigeria	To assess the factors contributing to the inequality in IPTp-SP use among pregnant women in Nigeria	Quantitative / Pregnant women	Significant inequality exists between pregnant women in IPTp-SP utilization as women from the wealthiest households recorded a higher uptake than women from poorer families. Free IPTp-SP and community-based delivery approach may help reduce the inequality, ultimately improving IPTp-SP uptake
Ndu, Mbachu, Anitube, Ezeoke (2020)	43	Inequities in the use of sulphadoxine-pyrimethamine for malaria prophylaxis during pregnancy in Nigeria	To highlight the geographic and socioeconomic variations and inequities in accessing and using SP for malaria prophylaxis in pregnancy, as well as client-related and service delivery determinants in Nigeria.	Quantitative / Pregnant women	The use of SP for MiP control is generally low across sub-population groups, including uneducated women, rural dwellers, women from the poorest households, and teenagers of reproductive age in Nigeria.
Noguchi, Grenier, Kabue, Ugwa, Oyetunji, Suhowatsky, Onguti, Orji, Whiting- Collins, Adetiloye (2020)	61	Effect of group versus individual antenatal care on uptake of intermittent prophylactic treatment of malaria in pregnancy and related malaria outcomes in Nigeria and Kenya: analysis of data from a pragmatic cluster randomized trial	To determine whether women randomized to group antenatal care (G-ANC) versus standard antenatal care (ANC) differed in IPTp uptake and insecticide-treated nets (ITN) use	Quantitative / Pregnant women	G‑ANC service delivery platform may support better ANC retention and increase in IPTp‑SP uptake
Nyaaba, Olaleye, Obiyan, Walker, Anumba (2021)	7	A socio-ecological approach to understanding the factors influencing the uptake of intermittent preventive treatment of malaria in pregnancy (IPTp) in South- Western Nigeria	To explore the factors contributing to the poor coverage of ITN and IPTp within a pluralistic health sector in Ogun State, southwest Nigeria	Qualitative / Pregnant women, ANC providers (TBA, faith-based birth attendants, public healthcare providers), community leaders	HCPs should network with and educate TBAs, and faith-based providers on MiP and IPTp-SP as this may improve pregnant women's access to and utilization of IPTp-SP towards MiP control
Okedo-Alex, Akamike, Alo, Agu, Nzeh, Ndukwe, Okoro, Abateneh, Uneke (2020)	58	Reaching the unreached: effectiveness and satisfaction with community-directed distribution of sulfadoxine- pyrimethamine for preventing malaria in pregnancy in rural South-East, Nigeria	To determine satisfaction with and effectiveness of community-directed distribution of IPTp-SP on uptake among pregnant women in Ebonyi State, Nigeria	Quantitative / Pregnant women	Community‑directed distribution of IPTp‑SP with active community engagement improved IPTp‑SP uptake and ITN use, in addition to increasing ANC utilization
Okeibunor, Orji, Brieger, Ishola, Otolorin, Rawlins, Ndekhedehe, Onyeneho, Fink (2011)	57	Preventing malaria in pregnancy through community-directed interventions: evidence from Akwa-Ibom State, Nigeria	To determine the degree to which community-directed interventions can improve access to malaria prevention in pregnancy	Quantitative / Pregnant women	Adequate access to malaria prevention remains low due to limited health systems capacities and lack of knowledge and resource demand at the household level. Community-based programs may be a cost-effective way to increase the uptake of malaria prevention like IPTp- SP
Olugbade, Ilesanmi, Gubio, Ajayi, Nguku, Ajumobi (2019)	44	Socio-demographic and regional disparities in utilization of intermittent preventive treatment for malaria in pregnancy - Nigeria demographic health survey 2013	To identify the factors determining uptake of IPTp-SP in Nigeria using the 2013 Nigeria Demographic Health Survey	**Quantitative / Women of child- bearing age**	Late initiation of IPTp-SP after the second trimester, due to late registration and inconsistent ANC attendance, was a contributory factor for poor SP utilization
Onwujekwe, Soremekun, Uzochukwu, Shu, Onwujekwe (2012)	6	Patterns of case management and chemoprevention for malaria-in-pregnancy by public and private sector health providers in Enugu state, Nigeria	To assess the knowledge and attitude of providers about malaria-in- pregnancy and compare the extent of provision of chemotherapy and chemoprevention (including IPTp) in public and private hospitals	**Quantitative**/ Doctors, pharmacists, and nurses	HCPs Low knowledge level about current best practices for MiP control, poor implementation of DOTs strategy, and lack of disposable cups and potable water in clinics for SP administration were barriers to SP uptake
Onyeaso & Fawole (2007)	22	Perception and practice of malaria prophylaxis in pregnancy among health care providers in Ibadan	To assess the knowledge of health care providers in Ibadan, south- western Nigeria on current concepts on malaria prophylaxis in pregnancy to ascertain their preferred drugs and to identify strategies that would enhance programmes for preventing malaria during pregnancy	**Quantitative**/Healthcare providers	Several knowledge gaps on current MiP prevention strategies among HCPs, including prescription of inefficacious drugs like chloroquine, are significant obstacles to SP uptake
Onyeneho, Idemili- Aronu, Igwe, Iremeka (2015)	32	Perception and attitudes towards preventives of malaria infection during pregnancy in Enugu State, Nigeria.	To explore and document perceptions and attitude associated with uptake of interventions to prevent malaria in pregnancy infection during pregnancy in Enugu State, Nigeria	Qualitative / Health workers, mothers and their husbands, grandmothers	Misconceptions about pregnancy and risk-benefit of SP and poor attitude of HCPs towards pregnant women are barriers to IPTp-SP uptake
Oyefabi, Sambo, Sabitu (2015)	19	Effect of primary health care workers training on the knowledge and utilization of intermittent preventive therapy for malaria in pregnancy in Zaria, Nigeria	To determine the effects of training primary health care workers on the utilization of IPTp among pregnant women who attend antenatal clinics in Sabon-Gari local government area (LGA) of Kaduna State, Nigeria	Mixed methods / Pregnant women	SP utilization was majorly affected by poor knowledge of HCPs, SP shortage and cost of SP, late ANC registration, and pregnant women's perceived risk-benefit of SP
Peters & Naidoo (2020)	49	Factors influencing the use of intermittent preventive treatment of pregnant women seeking care at primary healthcare facilities in the Bwari Area Council, Abuja, Nigeria	To explore the knowledge and practices of healthcare workers on the direct observation of IPT-SP amongst pregnant women attending antenatal care (ANC) in the Bwari Area Council (BWAC) of the Federal Capital Territory, Abuja, Nigeria	Qualitative / Healthcare workers	IPTp-SP uptake was hindered by poor adherence to the DOTs scheme, HCPs low awareness and understanding of the WHO guidelines on IPTp-SP, inadequate staff strength, lack of commodities including SP, potable water and disposable cups for SP administration
Pons-Duran et al (2020)	46	Coverage of intermittent preventive treatment of malaria in pregnancy in four sub-Saharan countries: findings from household surveys	To estimate the baseline coverage of IPTp in the countries' study areas, in addition to assessing the main factors affecting the uptake of IPTp	Quantitative / Women of reproductive age	ANC attendance and IPTp uptake was negatively affected by poor infrastructures at the health facility (e.g. lack of water), SP stock-outs, and negative attitudes of HCPs towards providing IPTp-SP
Tobin-West & Asuquo (2013)	37	Utilization of intermittent preventive treatment of malaria by pregnant women in rivers state, Nigeria	To assess the level of intermittent preventive treatment of malaria in pregnancy (IPTp) and to identify obstacles prohibiting its widespread use in Rivers state, Nigeria.	Quantitative / Pregnant and postnatal women	Misconceptions about IPTp-SP among pregnant women and persisting chloroquine dispensing instead of SP are major challenges to IPTp-SP uptake
